# Supplementary figures and images for: PqsE Is Essential for RhlR-Dependent Quorum Sensing Regulation in Pseudomonas aeruginosa
Source: mSystems. 2020 May 26;5(3):e00194-20. doi: 10.1128/mSystems.00194-20 (PMC7253366; doi:10.1128/mSystems.00194-20)

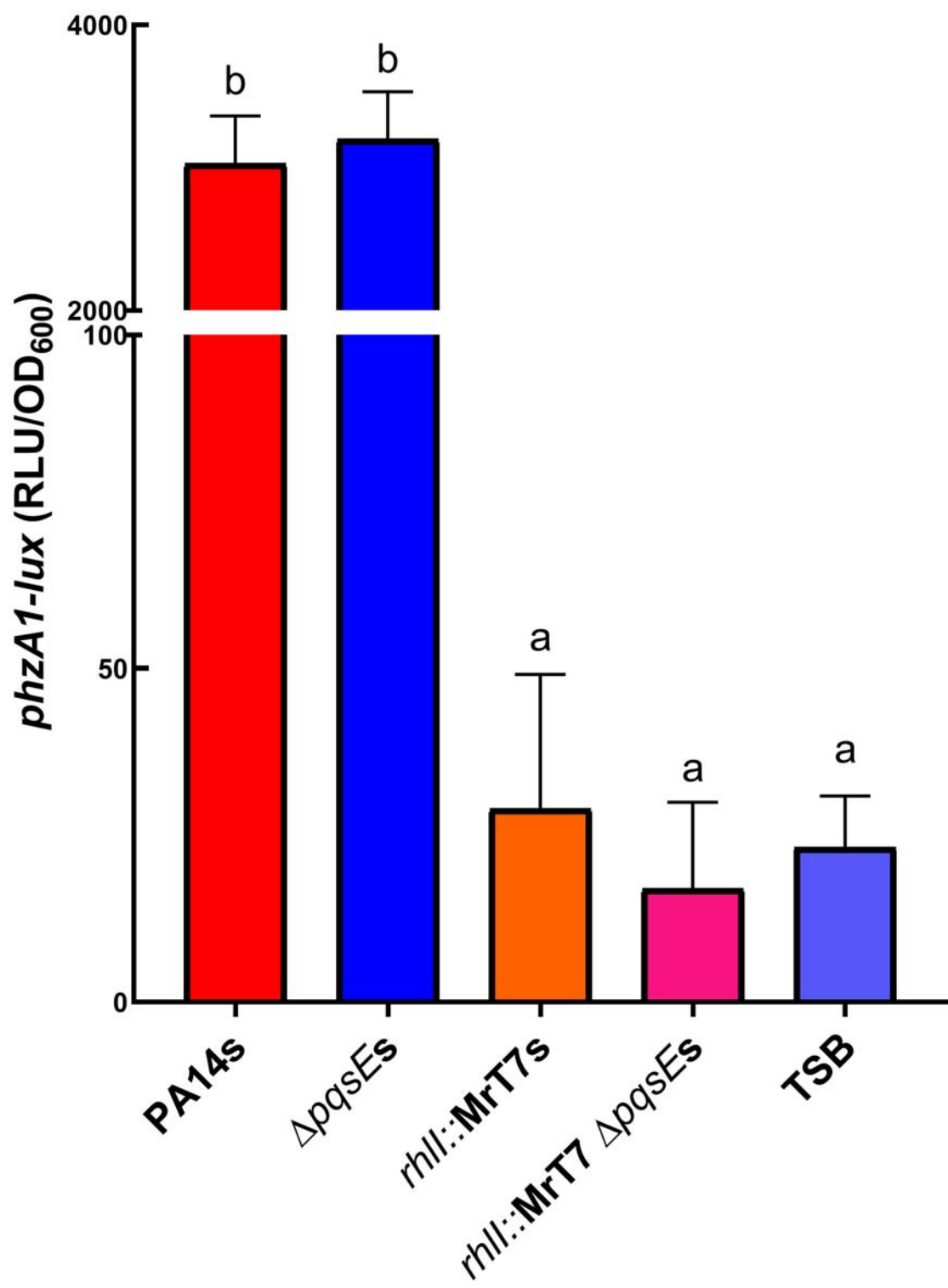

Supplement: FIG S1 [file mSystems.00194-20-sf001.pdf]

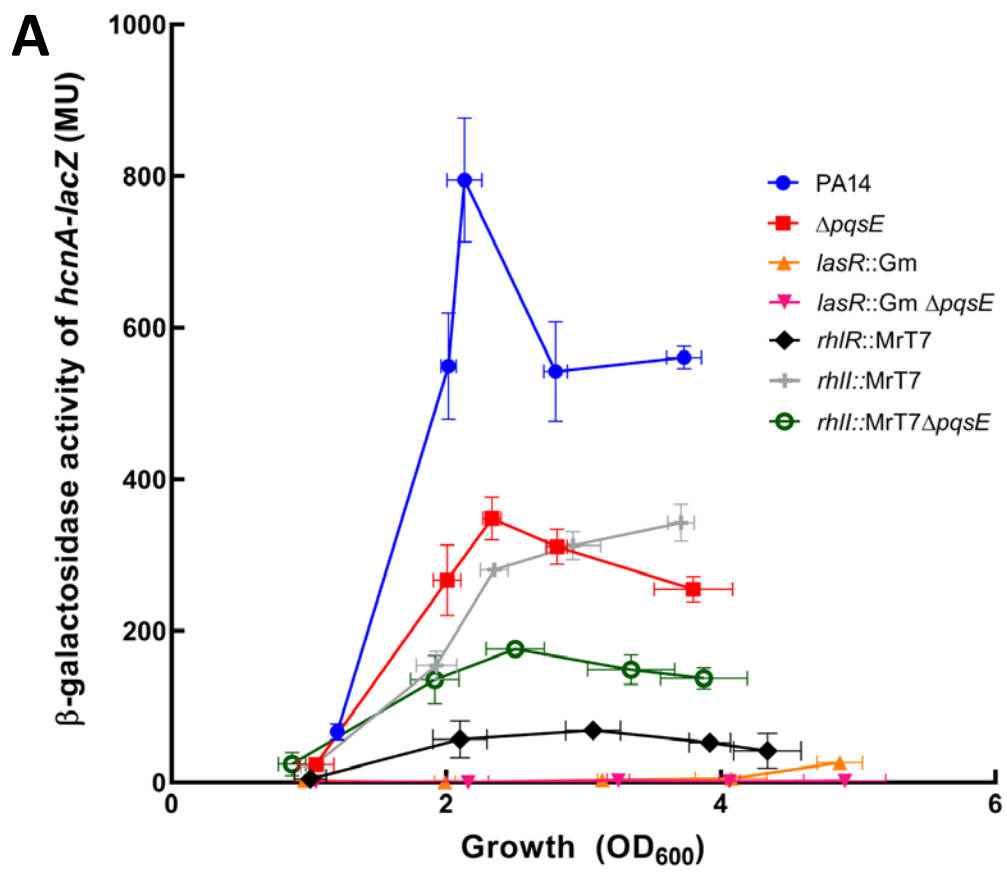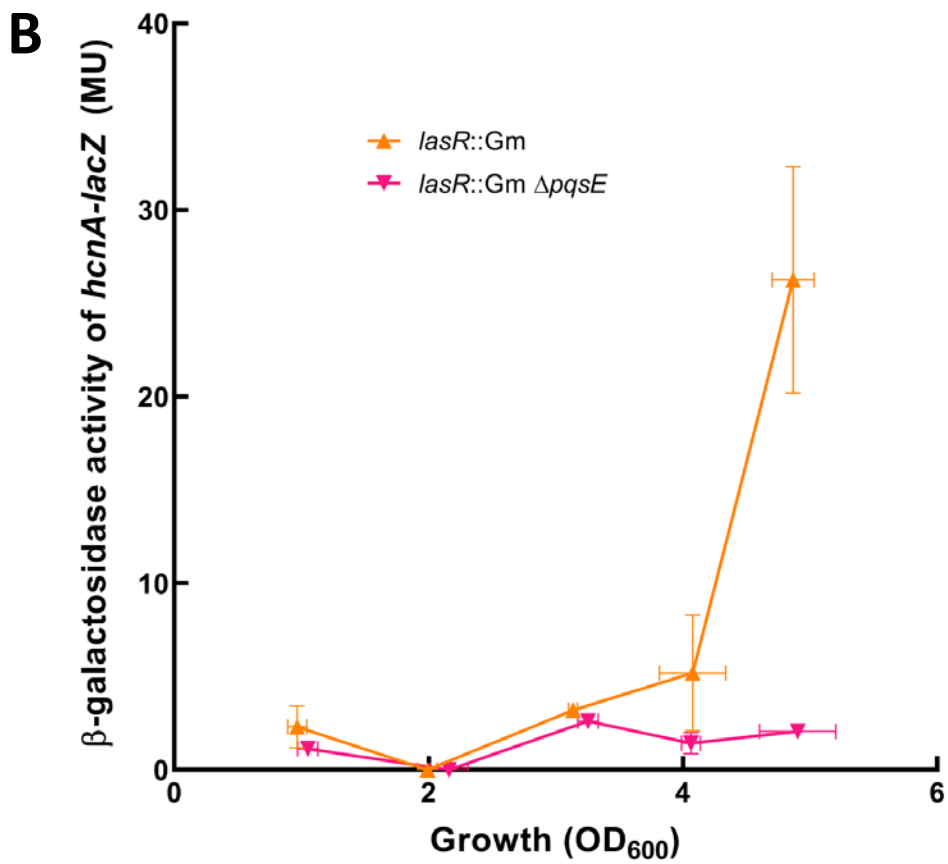

Supplement: FIG S2 [file mSystems.00194-20-sf002.pdf]

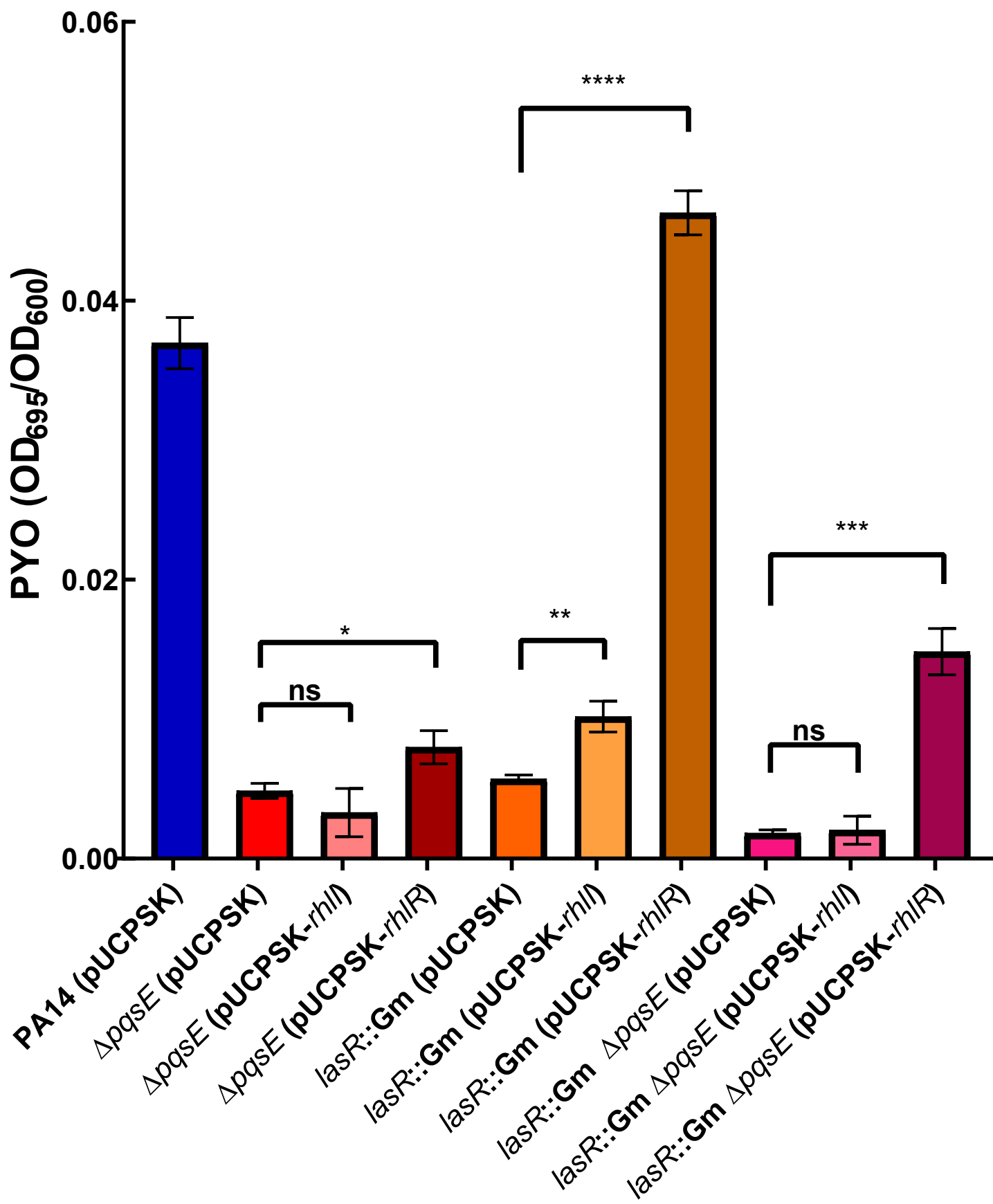

Supplement: FIG S3 [file mSystems.00194-20-sf003.pdf]
